# Supplementary material for: Cell lineage-specific transcriptome analysis for interpreting cell fate specification of proembryos
Source: Nat Commun. 2020 Mar 13;11:1366. doi: 10.1038/s41467-020-15189-w (PMC7070050; doi:10.1038/s41467-020-15189-w)
Supplement: Supplementary file 1 — Supplementary Information [file 41467_2020_15189_MOESM1_ESM.pdf]

Cell lineage-specific transcriptomes analysis for interpreting cell fate specification of proembryos

Zhou *et al.*

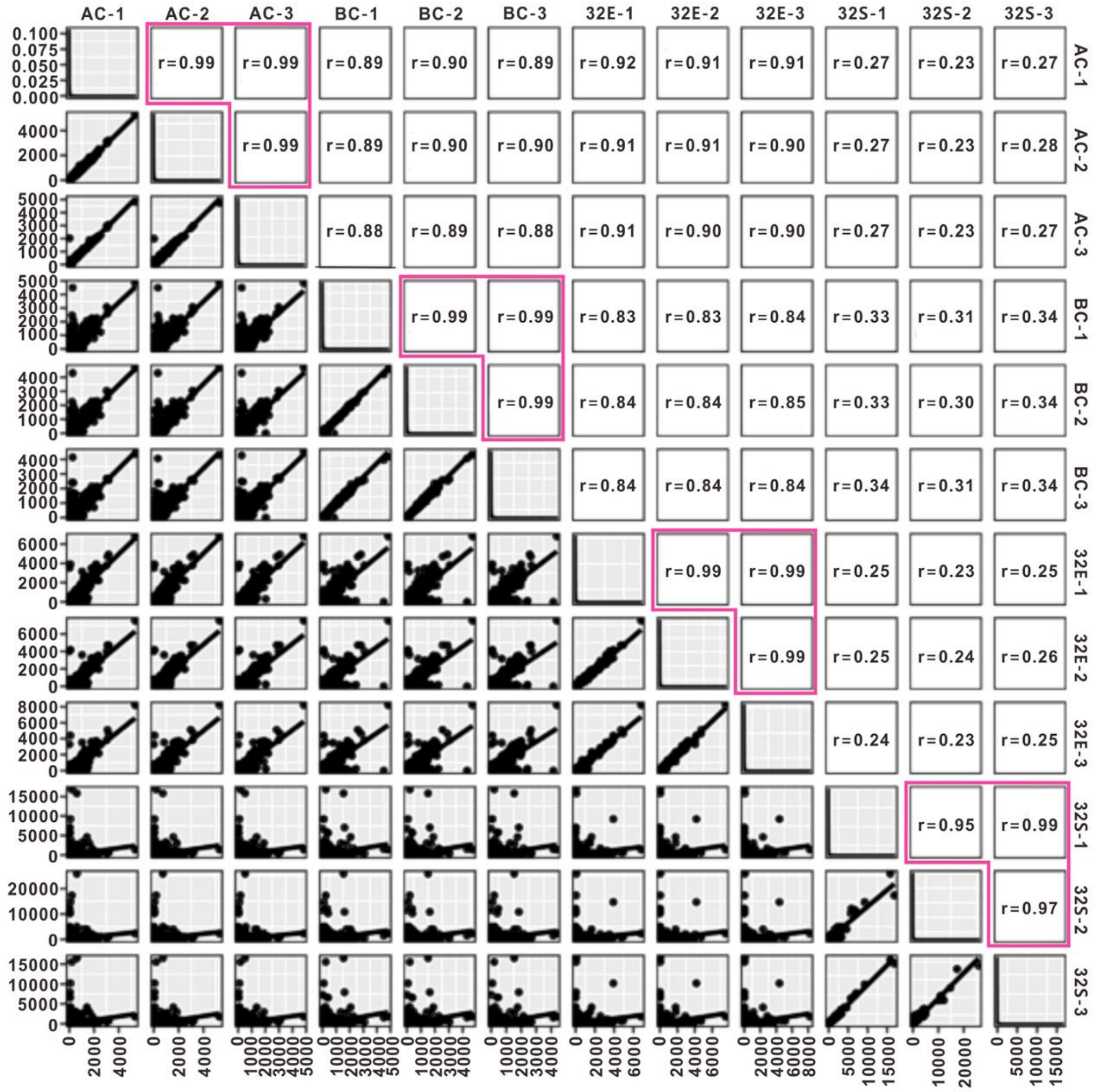

Supplementary Fig. 1 RNA-seq data from three biological replicates of each cell type are highly correlated.

The correlation coefficients between different samples are calculated according to the Pearson's correlation coefficient method. Three biological replicates of each cell type are delineated using magenta lines. AC, Apical cell; BC, Basal cell; 32E, embryo proper of 32-cell embryo; 32S, suspensor of 32-cell embryo. Same abbreviations were also used in the following figures.

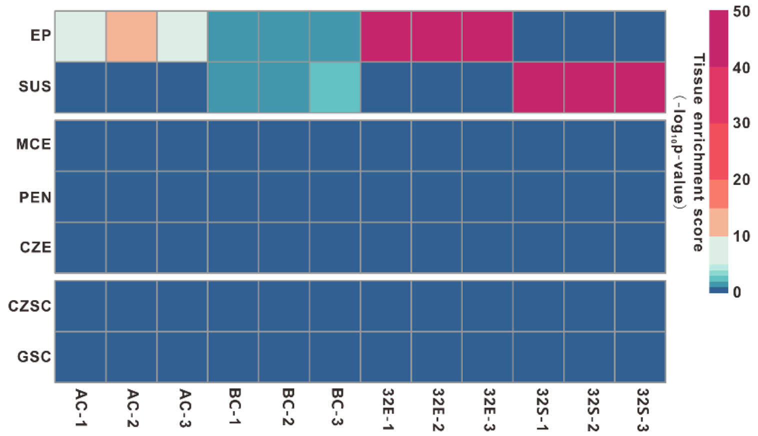

Supplementary Fig. 2 Detection of RNA contamination in each transcriptome.

Heat map displaying the results of tissue enrichment test of the transcriptomes of AC, BC, 32E and 32S. EP, embryo proper; SUS, suspensor; MCE, micropylar endosperm; PEN, peripheral endosperm; CZE, chalazal endosperm; CZSC, chalazal seed coat; GSC, general seed coat.

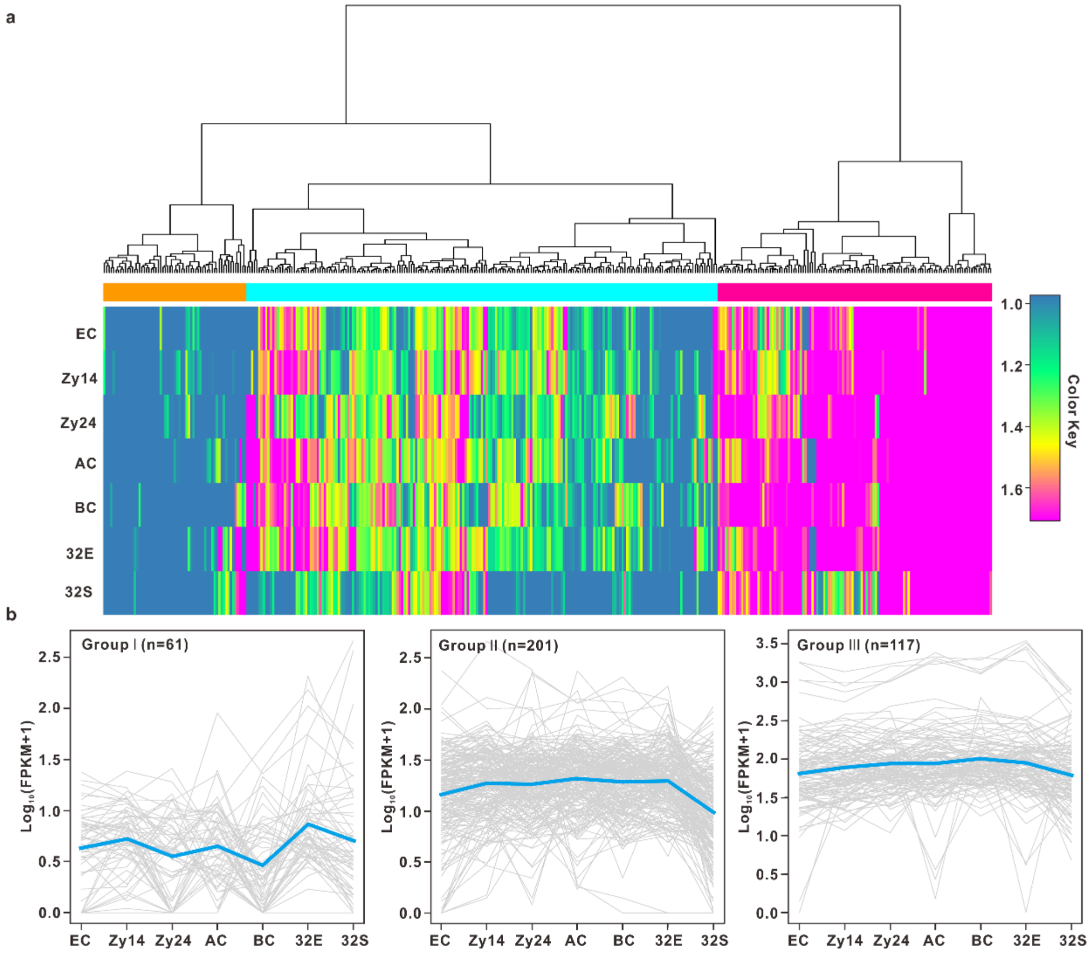

Supplementary Fig. 3 Hierarchical clustering of *EMBRYO-DEFECTIVE* (*EMB*) genes in different cell types.

a Heatmap showing the clustering results of *EMB* genes. **b** Expression profiles of the *EMB* genes in the egg cell, zygotes, apical and basal cell lineages of early proembryos. Blue line in each plot indicates the mean expression level of *EMB* genes.

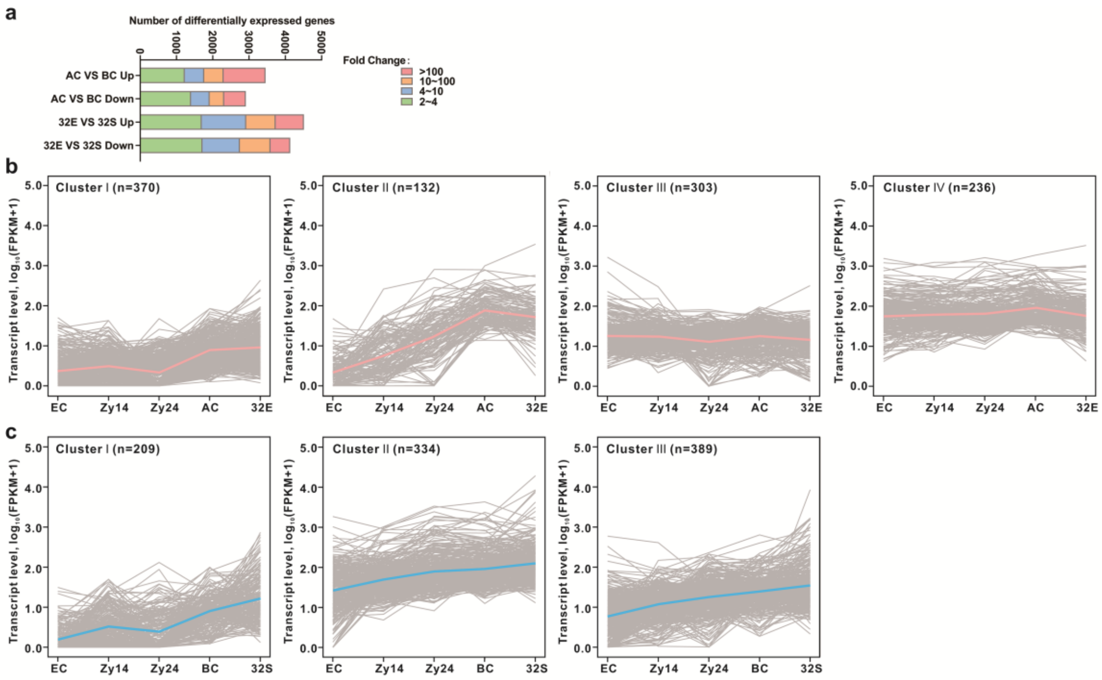

Supplementary Fig. 4 Expression profile of cell lineage-maintained genes during early embryogenesis.

a Graph showing the number of differentially expressed genes between apical and basal cell lineages at 1-cell and 32-cell embryo stage. **b** Expression profiles of the apical cell lineage- maintained genes in egg cells, zygotes, apical cell and embryo proper of 32-cell embryo. **c** Expression profiles of basal cell lineage-maintained genes in egg cells, zygotes, basal cell and suspensor of 32-cell embryo. Pink lines (**b**) and blue lines (**c**) indicate the mean expression level of all genes in different groups. EC, egg cell; Zy14, zygotes at 14 h after pollination; Zy24, zygotes at 14 h after pollination.

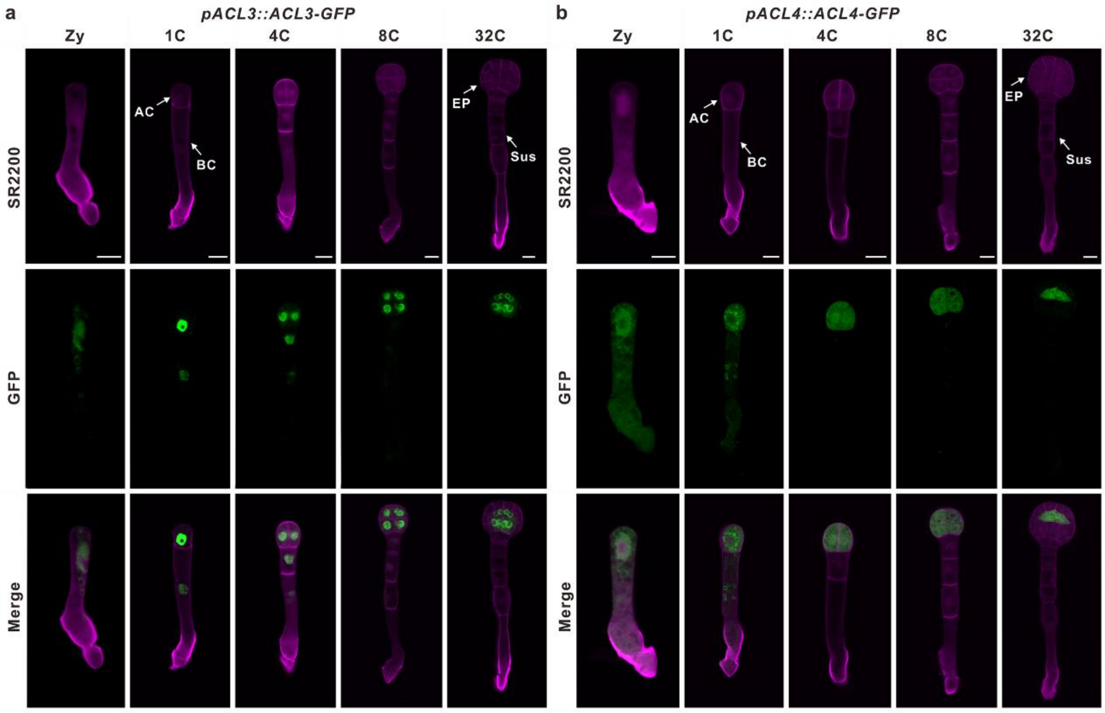

Supplementary Fig. 5 Expression pattern of selected apical cell lineage-maintained genes.

a, **b** Spatio-temporal expression of *ACL3* (**a**) and *ACL4* (**b**) in early embryogenesis. Bar=10 μm. Zy, zygote; 1C, 1-cell embryo; 4C, 4-cell embryo; 8C, 8-cell embryo; 32C, 32-cell embryo; AC, apical cell; BC, basal cell; EP, embryo proper; Sus, suspensor.

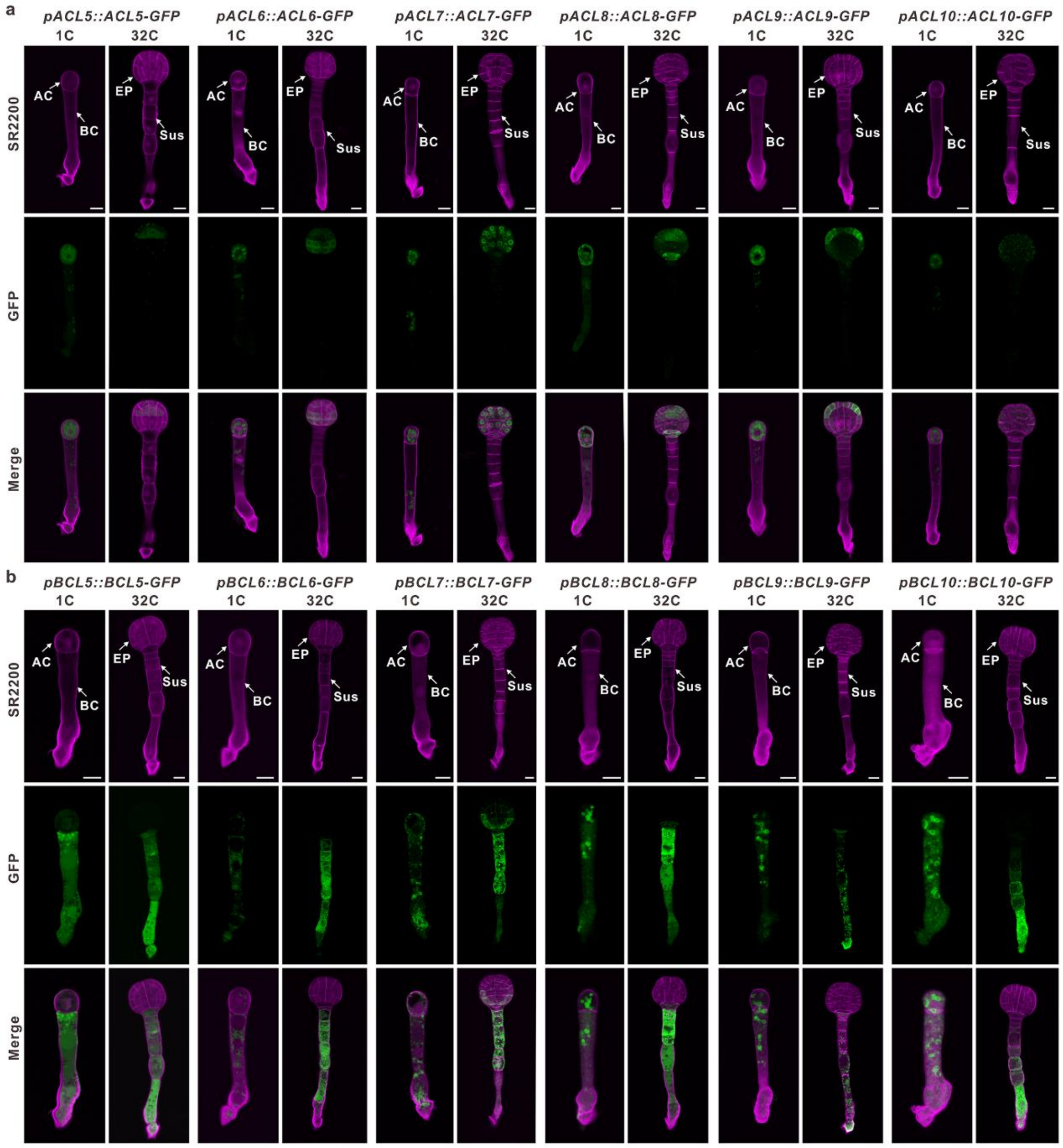

Supplementary Fig. 6 Expression pattern of selected cell lineage-maintained genes.

a, **b** Spatio-temporal expression of *ACL* (**a**) and *BCL* (**b**) marker genes in early proembryos at 1-cell and 32-cell embryo stage. Bar=10 μm. 1C, 1-cell embryo; 32C, 32-cell embryo; AC, apical cell; BC, basal cell; EP, embryo proper; Sus, suspensor.

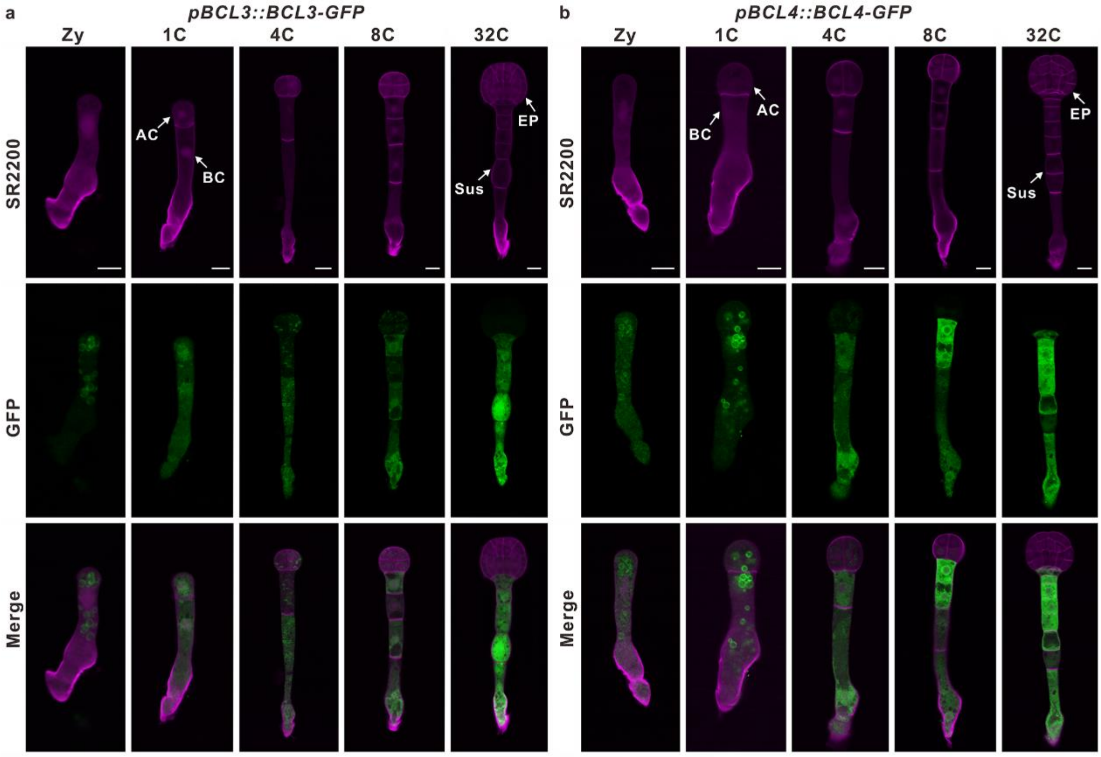

Supplementary Fig. 7 Expression pattern of selected basal cell lineage-maintained genes.

a, **b** Spatio-temporal expression of *BCL3* (**a**) and *BCL4* (**b**) in early embryogenesis. Bar=10 μm. Zy, zygote; 1C, 1-cell embryo; 4C, 4-cell embryo; 8C, 8-cell embryo; 32C, 32-cell embryo; AC, apical cell; BC, basal cell; EP, embryo proper; Sus, suspensor.

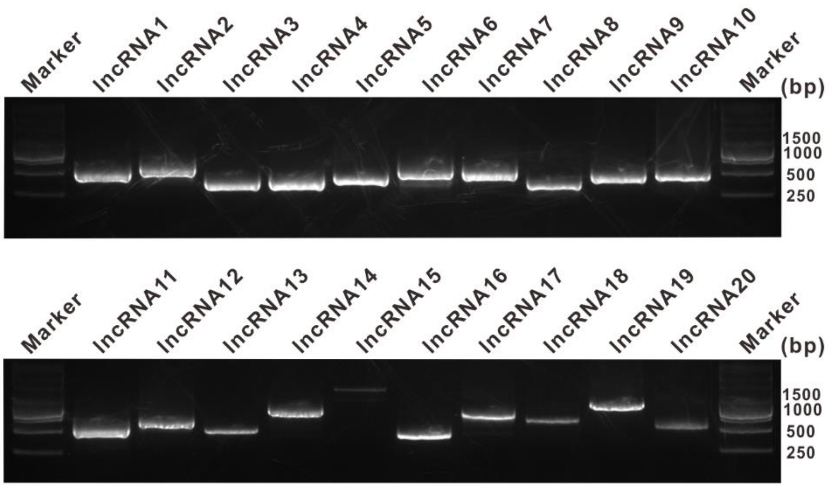

Supplementary Fig. 8 The expression of selected twenty lncRNAs in 32-cell embryos were confirmed by RT-PCR analysis.

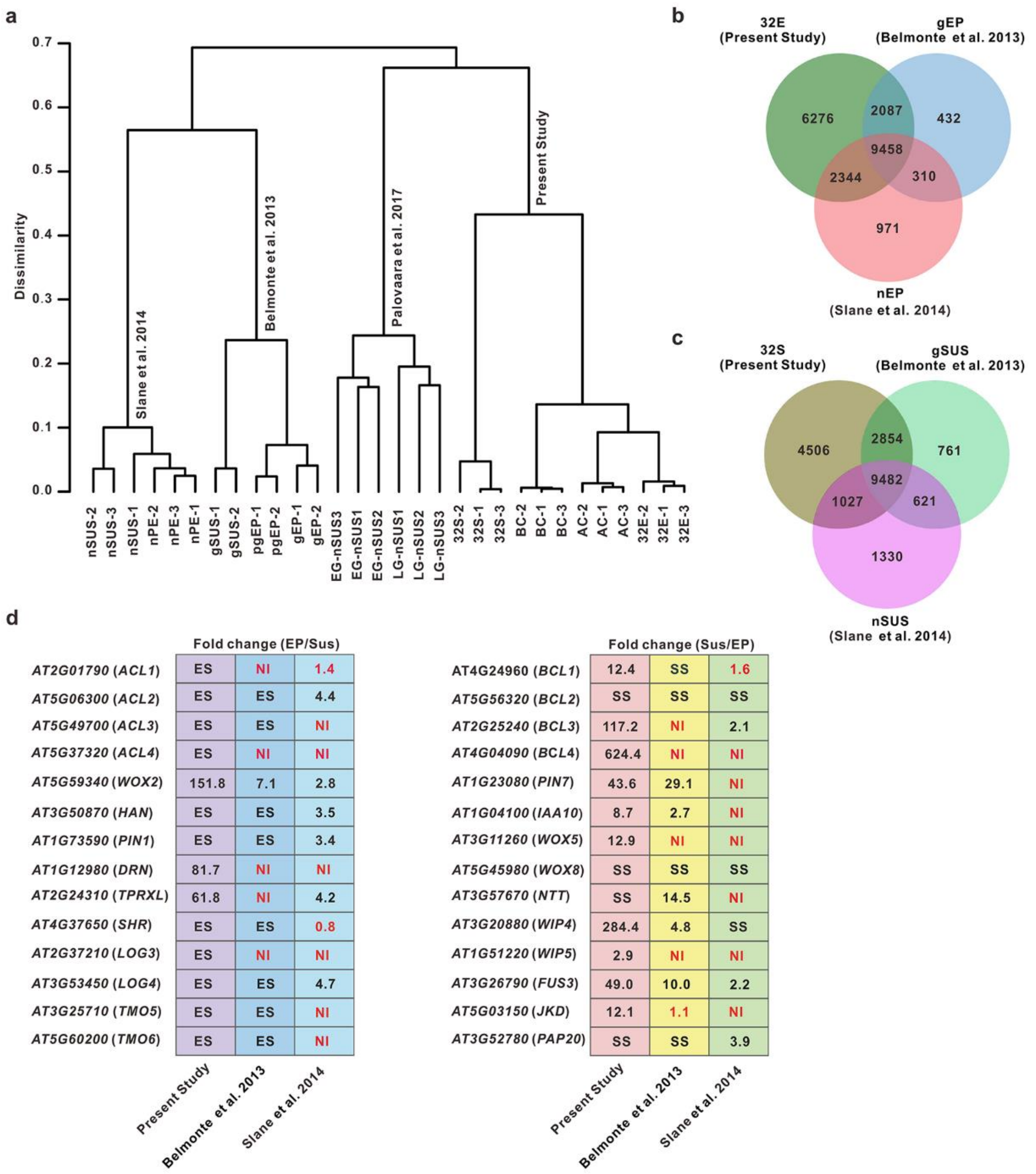

Fold change (EP/Sus)

	Present Study	Belmonte et al. 2013	Slane et al. 2014
AT2G01790 (*ACL1*)	ES	NI	1.4
AT5G06300 (*ACL2*)	ES	ES	4.4
AT5G49700 (*ACL3*)	ES	ES	NI
AT5G37320 (*ACL4*)	ES	NI	NI
AT5G59340 (*WOX2*)	151.8	7.1	2.8
AT3G50870 (*HAN*)	ES	ES	3.5
AT1G73590 (*PIN1*)	ES	ES	3.4
AT1G12980 (*DRN*)	81.7	NI	NI
AT2G24310 (*TPRXL*)	61.8	NI	4.2
AT4G37650 (*SHR*)	ES	ES	0.8
AT2G37210 (*LOG3*)	ES	NI	NI
AT3G53450 (*LOG4*)	ES	ES	4.7
AT3G25710 (*TMO5*)	ES	ES	NI
AT5G60200 (*TMO6*)	ES	ES	NI

Fold change (Sus/EP)

	Present Study	Belmonte et al. 2013	Slane et al. 2014
AT4G24960 (*BCL1*)	12.4	SS	1.6
AT5G56320 (*BCL2*)	SS	SS	SS
AT2G25240 (*BCL3*)	117.2	NI	2.1
AT4G04090 (*BCL4*)	624.4	NI	NI
AT1G23080 (*PIN7*)	43.6	29.1	NI
AT1G04100 (*IAA10*)	8.7	2.7	NI
AT3G11260 (*WOX5*)	12.9	NI	NI
AT5G45980 (*WOX8*)	SS	SS	SS
AT3G57670 (*NTT*)	SS	14.5	NI
AT3G20880 (*WIP4*)	284.4	4.8	SS
AT1G51220 (*WIP5*)	2.9	NI	NI
AT3G26790 (*FUS3*)	49.0	10.0	2.2
AT5G03150 (*JKD*)	12.1	1.1	NI
AT3G52780 (*PAP20*)	SS	SS	3.9

Supplementary Fig. 9 Comparison analysis of the nuclear and cellular transcriptome data of embryo proper and suspensor from different reports.

a Hierarchical clustering of the nuclear and cellular transcriptomes of embryo proper and suspensor from different reports. **b**, **c** Comparisons of detected genes in the transcriptome of embryo proper (**b**) and suspensor (**c**) from different reports. **d** Expression data (fold change) of genes with known expression pattern in the transcriptome from different reports. nSUS, suspensor nuclei; nPE, embryo proper nuclei; (GSE60242; Slane *et al.* 2014); gSUS, suspensor of globular embryo; gEP, embryo proper of globular embryo; pgEP, embryo proper of preglobular embryo; (GSE12404; Belmonte *et al.* 2013); EG-nSUS, early globular (EG)_suspensor nuclei; LG-nSUS, late globular (LG)_suspensor nuclei (GSE89101; Palovaara *et al.* 2017). EP, embryo proper; SUS, suspensor; ES, embryo proper specifically expressed gene; SS, suspensor specifically expressed gene; NI, not identified.

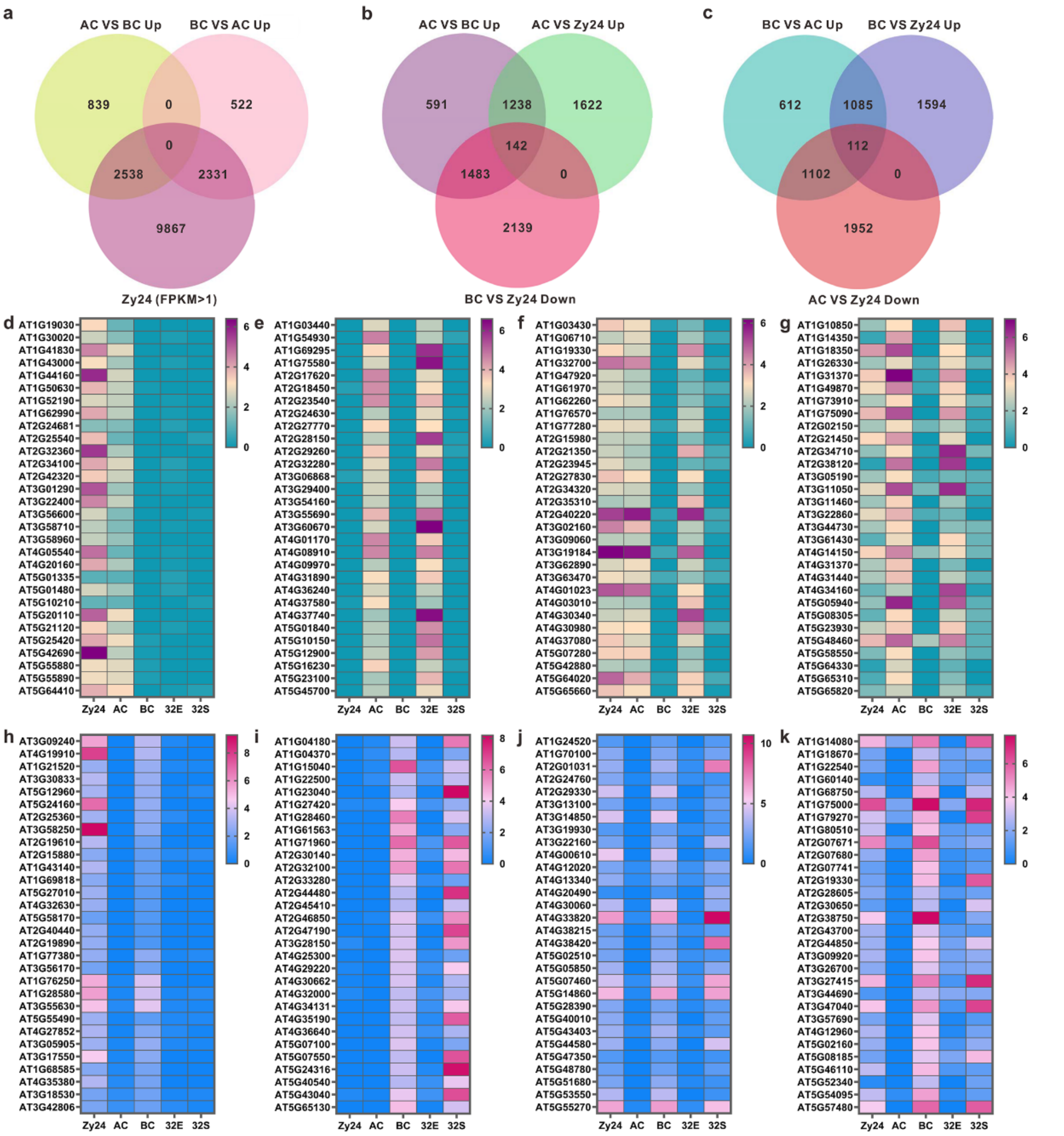

Supplementary Fig. 10 Expression pattern of differently expressed genes between apical and basal cell in early embryogenesis.

a Majority of upregulated genes in apical or basal cell are also expressed in the zygote. **b** Preferentially expressed transcripts in the apical cell are mainly resulted from *de novo* transcription in apical cell, downregulation in basal cell or both. **c** Preferentially expressed transcripts in the basal cell are mainly resulted from *de novo* transcription in basal cell, downregulation in apical cell or both. **d-g** Heatmap showing representative transcripts preferentially accumulated in the apical cell due to the uneven portion from the zygotes (**d**), specific *de novo* transcription in the apical cell (**e**), specific transcript degradation in the basal cell (**f**), both *de novo* transcription in the apical cell and specific degradation in the basal cell (**g**); **h-k** Heatmap showing representative transcripts preferentially accumulated in the basal cell due to the uneven portion from the zygotes (**h**), specific *de novo* transcription in the basal cell (**i**), specific transcript degradation in the apical cell (**j**), both *de novo* transcription in the basal cell and specific degradation in the apical cell (**k**).

Supplementary Table 1. RNA-seq library statistics of apical and basal cell lineages of early proembryos

Group	Sample	Raw reads pairs	Clean Reads pairs	Clean base (bp)	Total mapping rate (ratio)	Uniquely mapped (ratio)	Read pairs overlapping gene (ratio)	Expressed gene (FPKM>0)	Expressed gene rate (FPKM>0)	Expressed gene (FPKM>1)	Expressed gene rate (FPKM>1)	Expressed gene (FPKM>1)	Expressed gene rate (FPKM>1)
AC	AC-1	28,285,967	23,167,971	6,950,389,461	42,248,472(91.2%)	41,797,873(90.2%)	21,177,833(91.4%)	16,171	49.9%	14,562	45.0%	14,517	44.8%
	AC-2	25,316,559	20,580,397	6,174,117,422	37,362,266(90.8%)	36,956,858(89.8%)	18,710,733(90.9%)	15,821	48.8%	14,496	44.7%		
	AC-3	20,676,207	16,699,630	5,009,888,431	31,531,763(94.4%)	31,080,068(93.1%)	15,159,097(90.8%)	14,392	44.4%	13,555	41.8%		
BC	BC-1	27,573,319	21,336,556	6,400,965,076	37,809,563(88.6%)	37,361,084(87.6%)	18,700,997(87.7%)	14,122	43.6%	13,034	40.2%	13,703	42.3%
	BC-2	33,391,555	26,211,683	7,863,502,806	46,908,077(89.5%)	46,400,217(88.5%)	23,107,752(88.2%)	14,846	45.8%	13,636	42.1%		
	BC-3	26,587,371	20,524,352	6,157,303,881	36,765,125(89.6%)	36,372,087(88.6%)	18,068,116(88.0%)	14,984	46.3%	13,784	42.5%		
32E	32E-1	23,028,416	17,068,189	5,120,450,732	31,983,336(93.7%)	31,554,263(92.4%)	15,530,651(91.0%)	17,594	54.3%	14,796	45.7%	15,104	46.6%
	32E-2	24,319,595	19,173,404	5,752,020,541	35,357,493(92.2%)	34,906,457(91.0%)	17,505,648(91.3%)	19,114	59.0%	15,322	47.3%		
	32E-3	22,284,666	16,937,254	5,081,175,624	31,329,485(92.5%)	30,928,030(91.3%)	15,609,421(92.2%)	17,663	54.5%	14,932	46.1%		
32S	32S-1	22,541,867	15,390,683	4,617,204,387	27,810,897(90.3%)	27,625,093(89.7%)	13,519,060(87.8%)	16,637	51.4%	14,788	45.6%	15,030	46.4%
	32S-2	23,983,509	16,989,540	5,096,855,939	30,480,963(89.7%)	30,270,195(89.1%)	14,433,027(85.0%)	16,516	51.0%	14,850	45.8%		
	32S-3	30,799,456	22,794,519	6,838,353,839	40,595,550(89.0%)	40,292,627(88.4%)	20,101,166(88.2%)	17,320	53.5%	14,963	46.2%		

Supplementary Table 2. Primers used for vector construction

Gene ID	Name	Forward (5'-3')	Reverse (5'-3')
AT2G01790	*ACL1*	NNNGGTACCCCTCCATAGAGACTCCAACAGAAG	NNNCTCGAGGCGCAAGAACTCAAGCAGAGCATCCATG
AT5G06300	*ACL2*	NNNGGTACCACTCTAATGGTTTGCGTTGCTTTG	NNNCCTAGGCGTAGCGGTAGCTACCTCCGAACCCGG
AT5G49700	*ACL3*	NNNGGTACCCAACGGCATCGTCGTCTTCAATAACC	NNNCTCGAGGTATGGCGGTGGAGCTCTGGCTG
AT5G37320	*ACL4*	NNNGGTACCCTCACATTAGGTGTTGGGATTAGAGG	NNNCCTAGGCCCTTCCAGCTTCTTCGACAGCAA
AT1G19540	*ACL5*	NNNGGTACCGTGATCTTAGTGAAAATCAGAGTTCG	NNNCTCGAGGATAAACCGGTTAAGAAACTCATCAAC
AT1G02390	*ACL6*	NNNGGTACCCGTTACTAGATGAGTTCTCTGTTTTGG	NNNGCGGCCGCTTTTTTCTTGACAACTCCGTTATTACCGGCC
AT3G60670	*ACL7*	NNNGGTACCGACTTGAATACTCTGATAACTAA	NNNCCTAGGATAAAGCGGGGCGCGTTGCGGCGG
AT5G48350	*ACL8*	NNNGGTACCCGCAGGTCTAGTGACCATTCGTTGC	NNNCTCGAGACTTAAAATCTGAACATCCAACAAAATG
AT5G05940	*ACL9*	NNNGGTACCAAAACCCCTTTCAAATAAGTTGGAACG	NNNCCTAGGAGAGACAGTGTACTTTGGAGGACCCTTTTG
AT2G05420	*ACL10*	NNNGGTACCGCGTCTTACTTTGACTATGGGAAC	NNN CTCGAGGAAAACATTGTCTTCGTTCAAAGAGAG
AT4G24960	*BCL1*	NNNGAGCTCCCACTTTGATCATCTCTCCATGAATAC	NNNCTCGAGGTGACTGTGAGCCTCGTGTCCCTCC
AT5G56320	*BCL2*	NNNGGTACCCTTGAATTGATTAAAGTAACGTGCG	NNNGCGGCCGCCCTCTGAGCCCGGAACTGTTTTCCGG
AT2G25240	*BCL3*	NNNTCTAGAGAGAGAACTGGGAAGGAGAAGGGTATAATTG	NNNCTCGAGATGTTTTGAAGGATCAAGAACTTGACCC
AT4G04090	*BCL4*	NNNGAGCTCCTCAATGTGGAGATGTTATGAAGC	NNNCTCGAGATTACGAGGAAAAATAGCCCTCGTTATCTC
AT3G59480	*BCL5*	NNNGGTACCTTCTCAGTCTCATTCTTTCGCAGG	NNNCTCGAGGTTTCCTTTCAGGAGGCTCTGAACTTC
AT5G57240	*BCL6*	NNNGCGGCCGCCAAACTTAGTCTTATATCCAGATTTCACAC	NNNCCTAGGGAGAGGAACAACTATAGGAGCTCGAGG
AT1G13590	*BCL7*	NNNGAGCTCTCCTGATAAACCGGAGATGATTCATG	NNNTCTAGAAGGAGATAGGTTTAAATCTTGTGTATAG
AT5G16570	*BCL8*	NNNGGTACCCCTTGGCAATTTTTCAGGTTTCGG	NNNCTCGAGTGGTTTCCAAAGGATTGTGGATTCAGC
AT5G53870	*BCL9*	NNNGAGCTCAACAACTATGGTCTGGAAAGCGATAC	NNNCTCGAGAGCAAACATCAGAAAGGTAAAAGTCGC
AT1G04645	*BCL10*	NNNGGTACCAGGAAGAAGCCAGAGGTCAATG	NNNCTCGAGCTTCCAAGGAAAACATTTCGCC
