# Supplementary material for: Cell lineage-specific transcriptome analysis for interpreting cell fate specification of proembryos
Source: Nat Commun. 2020 Mar 13;11:1366. doi: 10.1038/s41467-020-15189-w (PMC7070050; doi:10.1038/s41467-020-15189-w)
Supplement: Supplementary file 3 — Description of Additional Supplementary Files [file 41467_2020_15189_MOESM3_ESM.pdf]

Description of Additional Supplementary Files

File Name: Supplementary Data 1
Description: Differentially expressed genes in the process of apical and basal cell lineage specification.

File Name: Supplementary Data 2
Description: List of EMBRYO-DEFECTIVE (EMB) genes analyzed in the present study.

File Name: Supplementary Data 3
Description: Differentially expressed genes between apical and basal cell lineages of early proembryos at different stages.

File Name: Supplementary Data 4
Description: List of apical and basal cell lineage- maintained genes.

File Name: Supplementary Data 5
Description: The sequences of novel lncRNAs.

File Name: Supplementary Data 6
Description: Confirmed sequences of twenty novel lncRNAs.

File Name: Supplementary Data 7
Description: List of TF genes identified in the apical and basal cell lineages of early proembryos.

File Name: Supplementary Data 8
Description: List of TFs and their potential target-genes identified in apical and basal cell lineages of early proembryos at different stages.

File Name: Supplementary Data 9
Description: List of differently expressed genes between apical and basal cell that are hardly detected in 32-cell embryos.
