# Supplementary material for: Cell lineage-specific transcriptome analysis for interpreting cell fate specification of proembryos
Source: Nat Commun. 2020 Mar 13;11:1366. doi: 10.1038/s41467-020-15189-w (PMC7070050; doi:10.1038/s41467-020-15189-w)
Supplement: Supplementary file 13 — Reporting Summary [file 41467_2020_15189_MOESM13_ESM.pdf]

nature research

Corresponding author(s): Peng Zhao

Last updated by author(s): Feb 17, 2020

Reporting Summary

Nature Research wishes to improve the reproducibility of the work that we publish. This form provides structure for consistency and transparency in reporting. For further information on Nature Research policies, see Authors & Referees and the Editorial Policy Checklist .

Statistics

For all statistical analyses, confirm that the following items are present in the figure legend, table legend, main text, or Methods section.

n/a	Confirmed	
[ ]	[x]	The exact sample size (*n*) for each experimental group/condition, given as a discrete number and unit of measurement
[ ]	[x]	A statement on whether measurements were taken from distinct samples or whether the same sample was measured repeatedly
[ ]	[x]	The statistical test(s) used AND whether they are one- or two-sided<br>*Only common tests should be described solely by name; describe more complex techniques in the Methods section.*
[x]	[ ]	A description of all covariates tested
[ ]	[x]	A description of any assumptions or corrections, such as tests of normality and adjustment for multiple comparisons
[ ]	[x]	A full description of the statistical parameters including central tendency (e.g. means) or other basic estimates (e.g. regression coefficient) AND variation (e.g. standard deviation) or associated estimates of uncertainty (e.g. confidence intervals)
[ ]	[x]	For null hypothesis testing, the test statistic (e.g. *F*, *t*, *r*) with confidence intervals, effect sizes, degrees of freedom and *P* value noted<br>*Give P values as exact values whenever suitable.*
[x]	[ ]	For Bayesian analysis, information on the choice of priors and Markov chain Monte Carlo settings
[x]	[ ]	For hierarchical and complex designs, identification of the appropriate level for tests and full reporting of outcomes
[ ]	[x]	Estimates of effect sizes (e.g. Cohen's *d*, Pearson's *r*), indicating how they were calculated

Our web collection on statistics for biologists contains articles on many of the points above.

Software and code

Policy information about availability of computer code

Data collection	RNA-seq data were generated from Illumina HiSeq X Ten platform with PE150 mode.
Data analysis	Cutadapt (v1.15), Bowtie 2, RSEM, DESeq2, Tophat2, Cufflinks (v2.2.1), Cuffmerge, CNCI (v2), CPC (v0.9-r2), PLEK (v1.2), CPAT (v1.2.2), pfam_scan (v1.6-1), ASTALAVISTA (v3.2), FunTFBS and R package softwares were used for data analysis. Contamination in the transcriptome data sets was analyzed using the publicly available software (https://github.com/Gregor-Mendel-Institute/tissue-enrichment-test). The in house scripts for alternative splicing analysis (https://github.com/frasergen-rna/AS-cmp) and TF analysis (https://github.com/frasergen-rna/TF_prediction) are publically available from GitHub. All other details of the code used in the study are available from the corresponding author upon request.

For manuscripts utilizing custom algorithms or software that are central to the research but not yet described in published literature, software must be made available to editors/reviewers. We strongly encourage code deposition in a community repository (e.g. GitHub). See the Nature Research guidelines for submitting code & software for further information.

Data

Policy information about availability of data

All manuscripts must include a data availability statement. This statement should provide the following information, where applicable:

- Accession codes, unique identifiers, or web links for publicly available datasets
- A list of figures that have associated raw data
- A description of any restrictions on data availability

All RNA-seq data have been uploaded to the NCBI Gene Expression Omnibus (GEO) under accession GSE135422. The source data underlying Figs. 2, 3, 6a-c, e, f, h-j, 7 as well as Supplementary Figs. 2, 3b, 4a-c, 8 and 10d-g, h-k are provided as a Source Data file.

Field-specific reporting

Please select the one below that is the best fit for your research. If you are not sure, read the appropriate sections before making your selection.

☒ Life sciences ☐ Behavioural & social sciences ☐ Ecological, evolutionary & environmental sciences

For a reference copy of the document with all sections, see nature.com/documents/nr-reporting-summary-flat.pdf

Life sciences study design

All studies must disclose on these points even when the disclosure is negative.

Sample size	Four different cell types including apical cell, basal cell, embryo proper and suspensor were collected for RNA-seq. And three independent biological replicates have been performed for each cell type. The transcripts from the same cell type but different biological repeats are highly correlated ($r \geq 0.95$). Hence, there are twelve independent transcriptome data of early embryos, which are sufficient for subsequent analysis.
Data exclusions	No data were excluded from the analysis.
Replication	The transcriptome from three independent biological replicates are highly correlated ($r \geq 0.95$).
Randomization	Three independent biological replicates were allocated into experimental groups according to the cell type.
Blinding	Since three independent biological replicates for each cell type were already allocated into the same group, blinding was not relevant to our study.

Reporting for specific materials, systems and methods

We require information from authors about some types of materials, experimental systems and methods used in many studies. Here, indicate whether each material, system or method listed is relevant to your study. If you are not sure if a list item applies to your research, read the appropriate section before selecting a response.

Materials & experimental systems

n/a	Involved in the study	
☒	☐	Antibodies
☒	☐	Eukaryotic cell lines
☒	☐	Palaeontology
☒	☐	Animals and other organisms
☒	☐	Human research participants
☒	☐	Clinical data

Methods

n/a	Involved in the study	
☒	☐	ChIP-seq
☒	☐	Flow cytometry
☒	☐	MRI-based neuroimaging
